# Statistical Detection of EEG Synchrony Using Empirical Bayesian Inference

**DOI:** 10.1371/journal.pone.0121795

**Published:** 2015-03-30

**Authors:** Archana K. Singh, Hideki Asoh, Yuji Takeda, Steven Phillips

**Affiliations:** 1 ATR Neural Information Analysis Laboratories, Kyoto 619-0288, Japan; 2 National Institute of Advanced Industrial Science and Technology (AIST), Tsukuba 305-8568 Japan; Universiteit Gent, BELGIUM

## Abstract

There is growing interest in understanding how the brain utilizes synchronized oscillatory activity to integrate information across functionally connected regions. Computing phase-locking values (PLV) between EEG signals is a popular method for quantifying such synchronizations and elucidating their role in cognitive tasks. However, high-dimensionality in PLV data incurs a serious multiple testing problem. Standard multiple testing methods in neuroimaging research (e.g., false discovery rate, FDR) suffer severe loss of power, because they fail to exploit complex dependence structure between hypotheses that vary in spectral, temporal and spatial dimension. Previously, we showed that a hierarchical FDR and optimal discovery procedures could be effectively applied for PLV analysis to provide better power than FDR. In this article, we revisit the multiple comparison problem from a new Empirical Bayes perspective and propose the application of the local FDR method (locFDR; Efron, 2001) for PLV synchrony analysis to compute FDR as a posterior probability that an observed statistic belongs to a null hypothesis. We demonstrate the application of Efron's Empirical Bayes approach for PLV synchrony analysis for the first time. We use simulations to validate the specificity and sensitivity of locFDR and a real EEG dataset from a visual search study for experimental validation. We also compare locFDR with hierarchical FDR and optimal discovery procedures in both simulation and experimental analyses. Our simulation results showed that the locFDR can effectively control false positives without compromising on the power of PLV synchrony inference. Our results from the application locFDR on experiment data detected more significant discoveries than our previously proposed methods whereas the standard FDR method failed to detect any significant discoveries.

## Introduction

A new class of simultaneous inference problems has emerged with the recent development in EEG technology, which provides us with means of acquiring thousands of signals in the form of large-scale high-dimensional data. Establishing functional connectivity inference in topological time-frequency maps, which is increasingly the focus of neuroimaging research in recent years, epitomizes a classical case of such problems [1, 2, 3, 4, 5, 6, 7]. In particular, there is a greater appreciation for the complex role that synchrony plays in cognitive tasks, and how synchrony varies along spatial, temporal and spectral dimensions. For instance, when the brain engages in visual search, it locks frontal and parietal regions at certain time-frequency interval, and the topological, temporal, and spectral instances of this locking vary depending on whether the search employs top-down control of attention, as reported in previous studies on monkeys by [8] and on humans by [9].

An appropriate quantification of such topologically, spectrally, and temporally locked functional connectivity requires state-of-art mathematical and statistical methods. Mathematically, several options exist, and the most commonly used option is a phase locking value, which is computed from wavelet decomposed EEG signals as the absolute average phase difference over trials between two signals [10]. PLV measures are applicable to non-stationary signals, though the phase and amplitude components may not be independent for Gaussian signals [11]. The absolute PLVs are bounded between 0 (no phase locking) and 1 (maximum phase locking). In event related EEG studies, the research question is often concerned with the changes in synchronization in response to a stimulus, therefore absolute PLV values are normalized with respect to a pre-stimulus baseline. Normalized PLVs are no longer bound between 0 and 1 and provide instantaneous measures of changes in phase coupling between two signals at any desired frequency and time. In this article, all references to PLV should be regarded as normalized PLV throughout the text unless stated otherwise. Statistically, quantifying the significant synchrony from PLV is difficult, because their high-dimensional time-frequency measurements from multiple sensors incur a serious multiple comparison issue. A common practice to address the problem is to restrict the hypothesis space, e.g., by averaging the data from the pre-specified sensors, time and frequency so that there is only one (or a few) summary statistic(s) per subject per condition. Although this is a fair and valid practice, it requires the region of interest to be defined a priori, which is often not available especially for EEG synchrony research problems.

High dimensional data pose several challenges to the conventional approach for multiple comparison correction procedures. The main problem with this approach is that it treats large-scale testing as a simple extension of single hypothesis testing, which by design aims at rejecting the null hypothesis with high probability using the data only from the tested individual hypothesis. On the contrary and in practice, large scale testing is pursued with a different scientific motivation. A case in point is the PLV analysis, where the results are plotted in a functional connectivity map, showing the interesting discoveries concerning the hypothesis under investigation. For instance, we may be interested in exploring where, when, and at what frequencies do we observe phase locking in the brain in response to the task under investigation. Here, we expect each dimension of hypothesis space to have a distinct dependence structure depending on the expected joint distribution of discoveries (non-nulls) and non-discoveries (nulls). Therefore, the single testing approach, that requires simply pooling all the hypotheses from different dimensions together to determine a common cutoff without accounting for the joint distribution of the hypotheses, tends to be highly insensitive.

The problems of identifying statistically significant functional connectivity in high-dimensional space-time-frequency synchrony data have been reported by various authors in neuroimaging literature [12, 13, 14, 15, 16, 17, 18, 19]. However, only a few studies have suggested the solutions to this problem. In addition to frequentist inference, Bayesian inference for modeling effective connectivity e.g. a dynamic causal model is also available for time-frequency EEG data, which does not require a correction of multiple comparison [20]. However, it requires the sensors of interest to be defined a priori [21]. In the context of fMRI imaging of brain activity, the multiple comparison issue was addressed by random field theory that statistically extracts and quantifies topological features of the SPM, e.g., number of regions, their spatial extent or peak height, and thus explicitly accounts for the dependence in neuroimaging data [16, 22, 23]. The use of permutation testing also improved the inference in fMRI studies to identify a cluster-level threshold [24]. For sparsely sampled EEG space-time-frequency data, both the topological and cluster-level inference require a priori knowledge about the region of interest in at least a single dimension. This restricts their applications to either space-time (when frequency band of interest is known a priori) or time-frequency (when sensor of interest is known a priori) analysis [15, 25, 26, 27].

Previously, we suggested two alternatives that avoid the difficulties with topological FDR just mentioned [17, 28]. One way is to organize hypotheses into a hierarchy of families. This approach is employed in the hierarchical false discovery rate (hFDR) procedure [29]. Another way is to modify the test statistic so that it explicitly shares the dependence information from across all the tests as suggested by [30] in his optimal discovery procedure (ODP). In this paper, we present a third alternative, which is motivated by Empirical Bayesian approach to control local false discovery rate [31]. We outline these approaches.

The hFDR method ameliorates this problem for testing multiple hypotheses that are naturally organized into a hierarchical structure [29]. Although we do not know in advance precisely which tests are dependent, domain knowledge, or the sorts of hypotheses we wish to entertain help determine a hypothesis-tree hierarchy. Suppose synchrony is known to localize within four frequency bands. Arranging 20 frequency-specific PLV tests into four families (one for each band) and treating each family as a single test explicitly assumes that each test within a family is dependent. Hence, our adjustment for effective significance of each test is reduced from 20 to 4, thus raising the sensitivity of our testing procedure. For each family-level test that exceeds threshold, the procedure is recursively applied. In practice, we found that hFDR was effective in detecting true effects without raising the level of false discoveries beyond the expected level with reference to high-dimensional PLV data [17]. The advantage of hFDR is that it allows for a hierarchical and multi-dimensional inference, e.g., when we seek to test the more detailed hypothesis of synchrony within a particular time band (within a particular frequency band). A potential difficulty with hFDR occurs when there is no natural or a priori reason for arranging multiple hypotheses into a particular hierarchy.

It makes sense to increase the significance of a test if there exist other tests with the similar results to maximize expected true positives for each fixed expected false positive. This is precisely the approach that is followed in ODP [30] and locFDR [31] procedure, neither of which requires hierarchically organized families of hypothesis. ODP maximizes the number of expected true positives for each fixed number of expected false positives. The procedure relies on a statistic specifically designed for multiple large-scale testing, which is computed as the ratio of the sum of the probability of a given test under the alternative distribution of each test to that of its null distribution. The relative significance of each test is also estimated with reference to the other tests. In this way, the relative significance of each observed statistic increases when multiple true positive results are likely to have similar values, e.g., when the tests are dependent. The estimation of ODP statistic is based on true null and alternate probability densities of the data. We developed an application of ODP and demonstrated its effectiveness in the context of high-dimensional EEG phase locking data [28]. The current ODP application is available only for the data that conforms to normal density, which holds reasonably well in the case of PLV data. However, for other applications of ODP, where distributional assumptions are unknown or different, it will take some substantial developments to arrive at a generally applicable set of methods.

The local FDR procedure offers an Empirical Bayesian perspective on the issue of multiple comparisons in high dimensional PLV data. The formulation of locFDR underlies an Empirical Bayesian model, where priors of the parameters are common among all hypotheses, and are hierarchically estimated from the data. Local FDR is then estimated as the posterior probability that a hypothesis is null given its observed statistics [31]. The distribution of local FDR is modeled using a mixture density, and the inference is obtained by comparing the density of the null to the mixture distribution for any given statistic.

The local FDR procedure leverages high-dimensionality and dependence in the data to increase the sensitivity of the large-scale inference. Dependence is incorporated by sharing common information across the hypotheses for reducing both Type I (false discoveries) and Type II (missed discoveries) errors. Unlike hFDR, local FDR does not require any specific hierarchy among the hypotheses. Local FDR is more accurate than ODP, and has greater utility, because it can also be applied to other commonly used statistics in neuroimaging studies, such as t-statistic, z-score, and chi-square. Local FDR is also more robust to changes in symmetry of the statistic (e.g. z-score computed from the normalized PLV data) than ODP.

In this study, we focus on the application of locFDR and demonstrate its validity using simulations and experimental data in comparison with the other large-scale testing methods, ODP, hFDR, and the conventional FDR. The simulations reveal that these methods are more or less compatible in their detection sensitivity and FDR control. In experiment analysis, the locFDR option reveals the maximum number of significant pairs of synchrony at a conventional FDR cutoff of 5% in the expected region of interest. To the best of our knowledge, locFDR, hFDR, and ODP are the only currently available techniques to assess the significance of synchrony for large-scale testing that do not require a priori knowledge about the expected regions of interest (i.e., in time, frequency, or space). Therefore, a comparative review of these will serve as a useful reference for research investigations on brain connectivity using high-dimensional data.

## Materials and Methods

This section includes an overview of the concepts related to the phase locking value, frequentist and Bayesian formulations of FDR, and local FDR. For the purpose of comparing local FDR with other frequentist large-scale approaches, hFDR and ODP, they are also described briefly in the end.

### Phase Locking Value (PLV)

Phase-locking value (PLV [10]) is used as a measures of synchrony. PLVs are computed from stimulus-locked EEG data for each trial as measures of synchronization between brain regions. The phase *ϕ*(*t*, *f*, *n*, *e*_*i*_) at time *t*, frequency *f*, trial *n* and electrode *e*_*i*_ was computed by first convolving the data with a complex Morlet wavelet, defined as

$$\begin{array}{c} w_{\left(t,f\right)}={\left(\sigma_{t}\sqrt{\pi }\right)}^{\frac{1}{2}}e^{-\frac{t^{2}}{2{\sigma^{2}}_{t}}}e^{2\pi ift} \end{array}$$ (1)

where *σt* = *FTR*/2*πf*. Following [10], FTR can be set as 7. The range of *f* can be specified as desired for the analysis. The absolute value of PLV for electrode pair (*e*_*i*_, *e*_*j*_) is computed as:

$$\begin{array}{c} PLV_{(t,f,e_{i},e_{j})abs}=\frac{1}{N}|\sum_{h=1}^{N}e^{\left(\phi \left(t,f,n,e_{i}\right)-\phi \left(t,f,n,e_{j}\right)\right)}| \end{array}$$ (2)

where N is the number of trials. The absolute value of PLV varies between 0 (random phase difference, no phase locking) and 1 (constant phase difference, maximum phase locking). For event related studies, PLVs can be normalized with respect to a baseline, e, g, 200 ms pre-stimulus time period. The normalized values, *PLV*(*t*, *f*, *e*_*i*_, *e*_*j*_)*norm*, can be computed for each 1 ms time point as

$$\begin{array}{c} PLV_{(t,f,e_{i},e_{j})norm}=\left(PLV_{\left(t,f,e_{i},e_{j}\right)}-\mu_{base}\right)/\sigma_{base} \end{array}$$ (3)

where *μ*_*base*_ and *σ*_*base*_ are the mean and standard deviation of PLVs over the baseline period. PLVnorm represents normalized changes from the average baseline PLV at a given frequency and time and are no longer bound between 0 and 1. The positive PLVnorm values indicate increased synchronization and the negative values indicate decreased synchronization. PLVnorm values can be tested for significance using a parametric test, e.g., z-test and t-test. As PLV datasets involve simultaneous testing of many time-frequency windows across many electrode-pairs, their significance should be adjusted using multiple comparison procedures.

### False Discovery Rate (FDR)

FDR is defined as the expected proportion of falsely rejected hypotheses among the rejected ones, which is zero if there are no discoveries [32]. Let *H*_1_, …, *H*_*m*_ denote the collection of null hypotheses corresponding to the p-values, *p*_1_, …, *p*_*m*_. The possible outcomes of *m* tests can be summarized as in Table 1: *V* and *R* represent the number of false positives and declared significant positives, respectively.

$$\begin{array}{c} FDR=E(\frac{V}{R}|R>0)Pr\left(R>0\right) \end{array}$$ (4)

**Table 1:** Variables associated with the number of true negatives (TN), false negatives (FN), false positives (FP) and true positives (TP), for multiple testing of m null hypotheses.

	Declared Non-significant	Declared Significant	Total
True H_0_	U (TN)	V (FP)	m_0_
False H_0_	T (FN)	S (TP)	m-m_0_
Total	m-R	R	m

### FDRBH Procedure for controlling False Discovery Rate

Benjamini and Hochberg [32] introduced a method for controlling FDR (henceforth referred to as FDRBH), which follows a *fixed error rate* approach, where FDR is fixed at a chosen level, and a rejection region is determined as follows. Let *p*_(*i*)_ and *α* denote the ordered p-values and the pre-specified error rate, respectively. Then the rejection region for controlling FDR can be determined as $\gamma =max\{p_{\left(i\right)}:p_{\left(i\right)}\leq \alpha \frac{i}{m}\}$, where all null hypotheses corresponding to *p*_(*i*)_ ≤ *γ* are rejected. This method controls FDR at level *π*_0_ *α*, where *π*_0_ = *m*_0_/*m* is the proportion of true null hypotheses. Therefore, when all the null hypotheses are true (*m*_0_ = *m*), FDR is controlled at level *α*, and when some of the null hypotheses are rejected (*m*_0_ < *m*), the procedure controls FDR at a level far below *α*. The power of an FDR controlling procedure can be improved by substituting the estimated value of *π*_0_.

### Positive False Discovery Rate (pFDR) and its estimation

Storey introduced an alternative measure and threshold for false discovery rate, *positive false discovery rate* (pFDR) and q-value [33]. pFDR is defined conditional on there being positive findings, i.e., at least one discovery. Unlike FDRBH procedure, pFDR estimates false discovery rate given a fixed rejection threshold. This affords Storey’s procedure higher sensitivity over BH procedure, because it computes pFDR by estimating *π*_0_ over the specified rejection region. Assume that hypotheses *H*_1_, …, *H*_*m*_ are tested using statistic *z*_1_, …, *z*_*m*_ with corresponding p-values *p*_1_, …, *p*_*m*_. Let us denote a true null (*H*_*i*_ = 0) as ‘null’. Then, pFDR can be defined for a given significance region *Z*:

$$\begin{array}{c} pFDR\left(Z\right)=E(\frac{V(Z)}{R(Z)}|R\left(Z\right)>0), \end{array}$$ (5)

where *V*(*Z*) = #{null|*z*_*i*_ ∈ *Z*} and *R*(*Z* = #{*z*_*i*_:*z*_*i*_ ∈ *Z*}) (see Table 1). The rejection region is determined from the observed p-values assuming that all null hypotheses are identical with an identical region Γ for all tests. Let Γ = [0, *γ*], where *γ* ∈ [0, 1], then we reject all the null hypotheses with p-values less than *γ*. Since *p*_*i*_ are uniformly distributed by assumption and *π*_0_ *m* p-values are expected to be null, a conservative estimate of *π*_0_ can be given as follows:

$$\begin{array}{c} {\hat{\pi }}_{0}\left(\lambda \right)=\frac{\#\{p_{i}>\lambda \}}{(1-\lambda )m} \end{array}$$ (6)

where, *λ* is a tuning parameter, 0 ≤ *λ* ≤ 1, and # indicates the number of times the condition within parentheses holds true.

### Bayesian and global interpretation of pFDR

In a Bayesian sense, pFDR measures the probability that a significant test is a true null hypothesis *pFDR*(*Z*) = *P*(null |*z*_*i*_ ≤ *Z*)or *P*(null |*z*_*i*_ ≥ *Z*) for the left-tailed or right-tailed test. This is a natural Bayesian analogue to p-value, p-value(*z*_*i*_) = *P*(*z*_*i*_ ≤ *Z*|null), and is also known as q-value(*z*_*i*_). The significance of the observed statistic with respect to pFDR is computed as q-value(*z*_*i*_) = *min*(*pFDR*(*Z*)). Even though q-values are specific to each test, pFDR is essentially computed over the entire rejection tail, and hence it is also referred to as *global FDR*.

### Empirical Bayesian estimation of local False Discovery Rate (locFDR)

Efron et al. proposed an Empirical Bayesian concept for estimating false discovery rate using the mixture model [31]. In this method, *π*_0_ can be estimated using the area around the peak of the theoretical or empirical null distribution. They defined local false discovery rate (locFDR) as the probability of a hypothesis being in the null group given an observed value of the test statistic. Let us suppose that the hypothesis *H*_*i*_ is tested using a z-value, *z*_*i*_ resulting in the p-value, *p*_*i*_. Assume that each outcome *z*_*i*_ belongs to either of the two possible classes, “not interesting” or “interesting” PLV effects, corresponding to whether *z*_*i*_ is generated according to the null or alternative hypothesis. Let *p*_0_ and *p*_1_ be the probability of *z*_*i*_ being a null or non-null with the densities, *f*_0_(*z*) and *f*_1_(*z*), respectively.

$$\begin{array}{c} p_{0}=\;\;\;P(\text{null}\;),\;\;\;\;\;\;\;f_{0}(z)=\;\text{density}\;\text{if}\;\text{not}\;\text{interesting}\;\text{effect}\\ p_{1}=\;P(\text{not-null}\;),\;\;\;\;f_{1}(z)=\;\text{density}\;\text{if}\;\text{interesting}\;\text{effect} \end{array}$$

Assuming both classes, we can define mixture density for z-values, and apply Bayes theorem to obtain posteriori probabilities as follows.

$$\begin{array}{c} f(z)=p_{0}f_{0}\left(z\right)+p_{1}f_{1}\left(z\right),\\ p_{1}\left(z\right)=P\left(\text{not-null}\;|z_{i}=z\right)=1-p_{0}\frac{f_{0}\left(z\right)}{f(z)},\\ p_{0}\left(z\right)=P\left(\text{null}|z_{i}=z\right)=p_{0}\frac{f_{0}\left(z\right)}{f(z)}. \end{array}$$ (7)

By definition, locFDR is *p*_0_(*z*), the posterior probability that a hypothesis given the observed value z is null. A full Bayesian analysis would require a priori estimation of *p*_0_, *p*_1_, *f*_0_ and *f*_1_. In the proposed Empirical Bayesian model, locFDR is estimated empirically from the observed large-scale data assuming that most of the tests belong to null cases (*π*_0_ ≥ .9). This is done by 1) estimating the *f*(*z*) from the observed statistics, z, which can be done by fitting a smooth curve $\hat{f}(z)$ to the histogram of z, e.g., using Poisson fit, 2) defining the null density *f*_0_(*z*) either theoretically or empirically, and 3) assuming *p*_0_ = 1 ensures a conservative control of false discoveries both in tail-area FDR and local false discovery rate [31, 32]. For better sensitivity (i.e., to avoid overestimation), ${\hat{\pi }}_{0}$ from Eq 6 is substituted in the above model for computing locFDR.

For detecting significance of PLV, we assume *f*_0_(*z*) to be standard normal density $\phi (z)=exp(-z^{2}/2)/\sqrt{2x}$ and *p*_0_ is estimated from the histogram data near z = 0. An R locFDR package is available for estimating local false discovery rate using empirically or theoretically defined null densities from http://cran.r-project.org/web/packages/locfdr/index.html [31].

### Optimal Discovery Procedure

The procedure is implemented by computing ODP statistic, which requires estimation of null and alternate density functions from the observed data [30]. Let *m*_0_ be the number of true null hypotheses. Let *f*_*j*_ and *g*_*j*_ represent the respective true null and alternative densities for the *j* ^*th*^ hypothesis, *j* ∈ {1, …, *m*}, for the observed PLV data, *x*_*i*_, from the *i* ^*th*^(*i* = 1, *m*) electrode pair. The PLV data *x*_*i*_ is evaluated at the estimated probability density functions for all electrode pairs. Here, we assume a normal density function *ϕ*(.|*μ*, *σ* ^2^) for PLV data.

$$\begin{array}{c} {\hat{S}}_{\mathrm{ODP}}\left(x_{i}\right)=\frac{\sum_{j=m_{0+1}}^{m}g_{j}\left(x_{i}|H_{1}\right)}{\sum_{j=1}^{m_{0}}f_{j}\left(x_{i}|H_{0}\right)} \end{array}$$ (8)

where $\left(\mu_{j1},{\sigma_{j1}}^{2}\right)$ and $\left(0,{\sigma_{j0}}^{2}\right)$ are the mean and variance under alternative and null distributions, respectively. The intuition behind ODP is that the relative significance of each observed *x*_*i*_ increases when multiple true positive results are likely to have similar values. It makes sense to increase the significance of a test if there exist other tests with the similar results to maximize expected true positives for each fixed expected false positive. For example, if *x*_*i*_ corresponds to a true alternative null hypothesis, then its density, $\phi (x_{i}|\mu_{i1},\sigma_{i1}^{2})$ will make a substantial contribution to *S*_*ODP*_(*x*_*i*_). Furthermore, if there are other true alternatives with *μ*_*i*1_ ≈ *μ*_*j*1_, then the likelihood of *x*_*i*_ under $\phi (x_{i}|\mu_{j1},\sigma_{j1}^{2})$ will also make a substantial contribution to *S*_*ODP*_(*x*_*j*_). This procedure is optimal in the sense that for any pre-specified false positive rate, the ODP will have maximum true positive rate, see for detailed proof [34].

### Hierarchical FDR

The detailed procedure for hierarchical FDR (hFDR) is described in [29], and in the context of PLV analysis in [17]. The hFDR procedure is implemented by organizing the hypotheses into a family-subfamily tree hierarchy, where each (sub)family is associated with a single hypothesis. For instance, the tests for PLV data are grouped into *M* frequency and *N* time families based on the frequency and time band associated with the test. The *M* frequency families constitute the first level of the hierarchy. In this case, there are *N* time subfamilies at the second level for each frequency family, and within each time subfamily are the test statistics, one for each electrode pair, at the third (lowest) level. The data associated with each test within a family is summarized (i.e., averaged) to become the data for that family and its associated single hypothesis. The testing begins at the first level by applying a single-sample t-test and FDRBH control to test the *M* hypotheses in frequency family. If any hypothesis is rejected, then testing continues by testing the corresponding time subfamily at the second level. This process continues by recursively checking each child hypothesis of a parent that was rejected, and terminating upon not rejecting any children.

The FDR bound on a hypothesis tree is defined recursively as the sum of the expected proportion of the number of false discoveries to total discoveries for each family. An approximate bound is estimated as $\text{bound}=q\;\delta \;\frac{N_{d}+N_{f}}{N_{d}+1}$, where *N*_*d*_ is the number of observed discoveries, *N*_*f*_ is the number of families tested, and *δ*, a multiplicative constant, is set to 1 [29]. This bound varies in an interval [*q*, 2*q*], where *q* is the expected FDR level. When the number of discoveries far exceeds the number of family tested, the hierarchical FDR bound converges to *q*.

### Simulation

The purpose of simulations was to access the specificity and sensitivity of locFDR for multidimensional EEG data. We assigned true PLV effects to particular frequency-time bands. Hence, we associated PLV differences (between conditions) with 2 frequency bands, 12 time bands, 10 participants, and 25 electrode pairs (i.e., all pairwise combinations of 5 frontal and 5 parietal electrodes) constituting a 2×12×10×25 array. For frequency-time windows containing significant effects, 10 out of 25 pairs were defined as truly significant. The proportion of electrode pairs with true synchronies, *π*_1_, varied from 8% to 48% over a range of time-frequency windows.

To simulate dependence in the signals of electrode pairs across the time windows for specific frequency bands, their PLVs were generated from a multivariate normal distribution with the parameters *μ* and ∑ = *σ* ^2^ *R*. The mean vector, *μ* was assigned positive PLV effects to represent the true positives pertaining to alternative hypotheses, and zero PLV effects for null hypotheses representing true negatives. The covariance matrix, ∑ = *σ* ^2^ *R* was constructed by assigning the variance, *σ* ^2^ and correlation matrix, *R*, which were extracted from a real PLV dataset.

We were also interested in comparing locFDR, ODP and hFDR methods on grounds of the false positives and false negatives that they incur. A direct comparison of these methods is difficult as each works on a distinct operating principle, using a different measure of false positives. Nevertheless, a comparative evaluation of their detection power and actual FDR incurred with synthetic data, where configurations of true discoveries is known, would be useful for exploring any specific conditions that may warrant the use of one method over the other. For ODP, the tuning parameter *λ* was automatically chosen using a bootstrap distribution with 100 resamples following [35]. For a detailed algorithm, refer to Appendix A in Singh et al. [28]. For hFDR analysis, we constructed a 3-level hierarchical FDR tree so as to cast the hypotheses belonging to the frequency dimension at the first level, those belonging to the time dimension at the second level, and to each electrode pair at the third level. For FDRBH, all 600(= 2×12×25) hypotheses were pooled in a single family. A 5% FDR threshold was used in locFDR, ODP, hFDR, and FDRBH. The simulations were performed in R. The numbers of detected discoveries, false positives, false negatives were obtained by averaging over 100 runs.

### Experimental data analysis

The experimental data was acquired from a previously published visual search study [9]. The purpose of the experiment was to test the hypothesis that top-down driven control of visual attention in humans is accompanied by frontal-parietal synchrony in the lower gamma-band. Top-down signals were induced using distractors that share a feature (e.g., color, or orientation) with the target, yielding a steep search slope (search time increasing with display set size)—inefficient search. Bottom-up signals were induced using distractors with no feature in common with the target, yielding a flat slope (search time independent of set size)–efficient search. Participants showed significantly greater synchrony between frontal and parietal electrodes in the lower gamma-band during inefficient than efficient search. These results supported the results from a monkey study of Buschman and Miller, who used spectral coherence to examine the synchrony between frontal and parietal local field potentials [8].

Following [9], the frequency, *f* ranged from 10 Hz to 58 Hz at intervals of 2 Hz for the calculation of absolute PLV (Eq 2), and the baseline period was from 200 ms to 0 ms prior to stimulus onset for the calculation of normalized PLV (Eq 3). Our region of interest was confined to 25 electrode pairs, i.e., five frontal electrodes (F7, F3, Fz, F4, F8) by five posterior electrodes (T5, P3, Pz, P4, T6), located according to the International 10–20 system. Frequency was partitioned into lower (22–34 Hz) and upper (36–48 Hz) gamma bands, corresponding to the original studies [8, 9, 17]. Time was partitioned into twelve 50 ms windows for the first 600 ms after stimulus (search display) onset. We applied locFDR, FDRBH, hFDR, and ODP using 5% and 10% thresholds. locFDR was performed using R locFDR package. The frequency-time maps of the resulting synchrony were plotted using matlab. The theoretical null was specified as N(0, 1) to estimate the *p*_0_.

The PLV dataset and matlab code used for our analysis can be downloaded from http://dx.doi.org/10.6084/m9.figshare.1309378.

## Results

### Simulation

**Fig 1:**
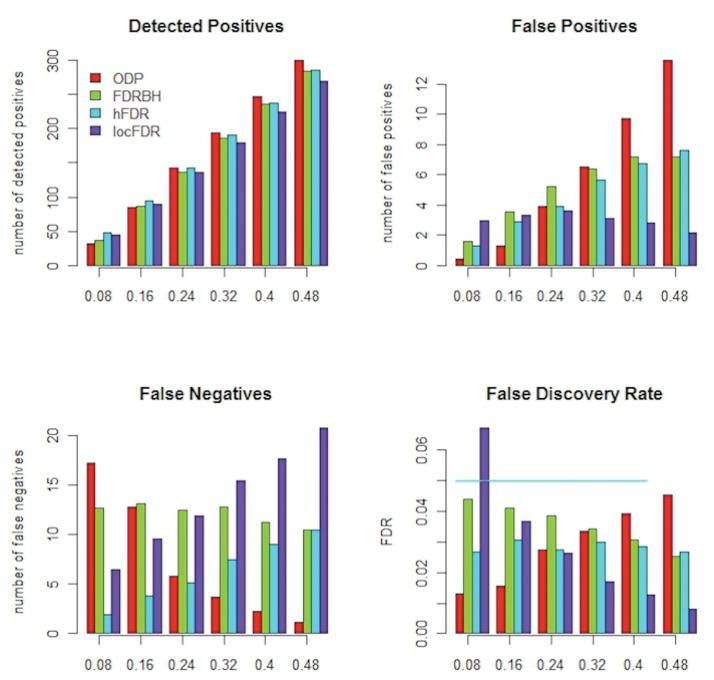
Comparison between FDRBH, hFDR, ODP, and locFDR. Each T-F window with assumed true synchrony effects has 10 pairs with true synchrony pairs; the x-axis represents the proportion of true positives; the graph panels represent: the total number of detected positives (top-left), the total number of false positives (top-right), the total number of false negatives (bottom-left), and false discovery rate (bottom-right).

The results of simulation analysis are summarized in Fig 1. As the proportion of true discovery, *π*_1_ = 1−*π*_0_, increases, locFDR exhibits a linear increase in the number of false negatives, a linear decrease in false discovery rate, whereas the number of false positives remains more or less constant. The local FDR was maintained well within the specified level of 5% except at extremely small proportion of true discovery; at *π*_1_ = 0.08, the locFDR was approximately 7%. ODP shows a linear increase in the number of false positives, a linear decrease in the number of false negatives, and a linear increase in FDR as the proportion of true discoveries increases. FDR was maintained within the specified level of 5% in all cases. For hFDR, there was a linear increase in both the number of false positives and false negatives, but FDR remained constant and below 5% in all the cases. While the detection power shows the same trend across the methods, the number of detected discoveries differs slightly depending on the configuration of true discoveries.

### Experiment

**Fig 2:**
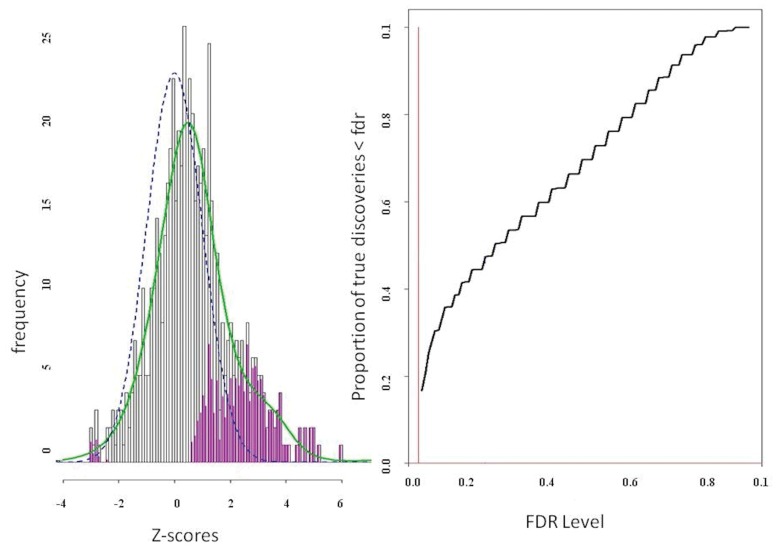
Empirical estimates of null and mixture density are shown on the left panel. The blue dashed line and green solid lines represents the null and mixture density, respectively. The pink bars indicate non-null counts. Power estimation as a function of estimated local FDR is shown on the right panel.

**Fig 3:**
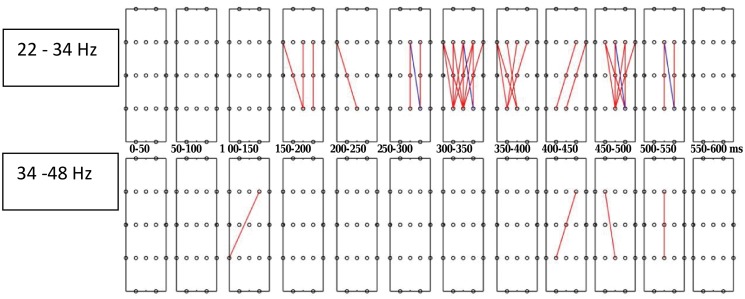
The synchrony map for experimental data from locFDR application. The top and bottom rows correspond to lower (22–34 Hz) and higher (36–48 Hz) gamma bands, and twelve 50 ms time windows spanning 600 ms post-stimulus duration within the band. The map shows 5 (frontal) × 5 (parietal) electrode pairs showing significantly greater phase-locking for the inefficient than efficient search conditions (red lines) for 5.

For the experimental data the null density, mixture density, and power (as a function of FDR) were estimated by locFDR (Fig 2). The locFDR method estimated 10% of the tests as belonging to the non-null group, that is, the proportion of pairs with true discovery (*π*_1_ = 1−*π*_0_), was estimated as 0.1. For comparison of locFDR results with those from hFDR, ODP and FDRBH, we first set the significant threshold at the conventional 5% level. The locFDR method detected 32 significant pairs (Fig 3). Most of the pairs are concentrated in the lower gamma band (22–34 Hz) over 300–500 ms post-stimulus time interval for significantly greater synchrony in the inefficient than efficient condition (red lines). The hFDR option detected 26 discoveries over 300–500 ms post-stimulus time interval in the lower gamma band (22–34 Hz) and none in upper gamma band (36–48 Hz) (see Fig 2 in [17]). ODP and FDRBH failed to detect any significant discoveries.

**Fig 4:**
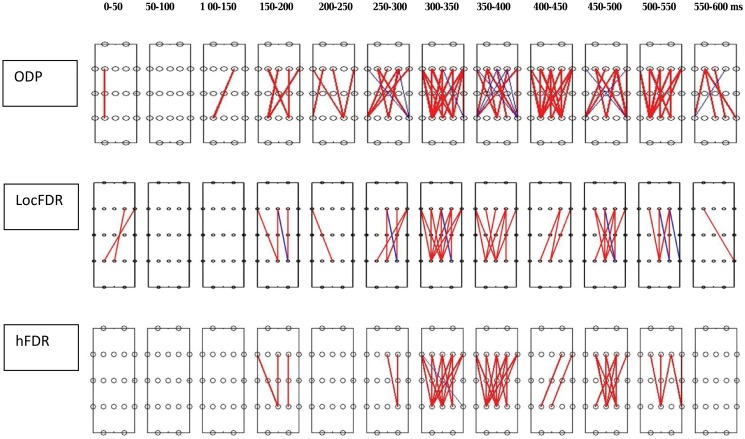
Synchrony maps from hFDR, ODP, and locFDR applications. Each map shows 5 (frontal) × 5 (parietal) electrode pairs corresponding to the lower (22–34 Hz) gamma band and twelve 50 ms time windows spanning 600 ms post-stimulus duration. The electrode pairs with significantly greater phase-locking for the inefficient than efficient search condition are indicated in red lines. The significant threshold is set at 10.

Next, we raised the significance threshold to 10% FDR level and obtained the results from all the four options. The results showed converging evidences of synchrony from locFDR, hFDR and ODP options predominantly in 22–34 Hz frequency band and 300–550 ms post stimulus time interval for the inefficient than efficient condition (Fig 4). The FDRBH option showed only 10 pairs with significantly greater synchrony in the lower gamma band for inefficient than efficient condition (results for FDRBH are not shown).

## Discussion

This article describes the application of a novel local false discovery rate procedure [31] as a Bayesian alternative to the problem of detecting statistically significant synchrony in large-scale testing of PLVs. The last couple of decades have witnessed a gradual paradigm shift in the way we interpret the significance from large-scale hypothesis testing, which has become pervasive in all neurophysiological applications. This shift began with the paper by [32], who showed that controlling false discovery rate instead of family-wise error rate would lead to higher detection power without compromising on Type I error control. Since then, there have been continuing efforts to improve the sensitivity of FDR controlling method. Several new approaches have been adopted for large-scale testing of high-dimensional neuroimaging data, e.g., including cluster-wise false discovery rate procedure [36], topological false discovery rate control [23], hierarchical false discovery rate control [17], optimal false discovery rate control [28] etc. among others.

EEG phase locking analysis involves a complex dataset with a small proportion of significant synchrony effects in multiple dimensions of time, frequency, and electrode space, each with a distinct dependence structure. The inference in classic hypothesis testing theory is essentially interpreted by thresholding p-values that are obtained individually from tail-area of the null distribution under each individual hypothesis, and FDRBH is no exception. Inferences derived from classical hypothesis tend to be too conservative to reveal even the true significant discoveries when applied for statistical testing of PLV.

There are several limitations with classic single hypothesis testing. The main drawback is that it prevents the dependence structure among the expected true discoveries to be incorporated in the simultaneous evaluation of significance from multiple hypotheses testing. The second limitation is due to the tail-area based testing; it may lead to a flawed inference when the distribution of the statistic is asymmetrical or bumpy. Another important issue concerns the interpretation of the p-value itself. Although a p-value tells us whether or not an observed statistic is significant, it does not tell us whether it is generated by the null or alternative distribution. For example, the p-value of z-statistic, *P*(*Z* ≥ 1.96|*null*) has a sensible interpretation, but *P*(*Z* = 1.96) = 0 is uninformative.

In our previous articles, we adopted hierarchical FDR method and optimal discovery procedures to overcome some of the problems as we just mentioned [17, 28]. The hFDR testing clusters the multi-dimensional distribution of PLVs into a single tree of nested families. The families that are likely to contain true negatives are excluded hierarchically, reducing the dimensionality. The remaining significant clusters provide a multi-scale representation of the PLV signal implicitly accounting for underlying dependence. ODP testing relies on a test statistic that is designed for multiple testing by explicitly sharing the dependence information from across all the tests, and it optimizes FDR by estimating expected true and false positives. The significance of ODP is tested using *pFDR* = *P*(*null*|*Z* ≥ *z*), which is like the p-value with a reversed conditioning. It can be estimated specific to each observed statistic (q-value) from the points falling in the entire tail-area, *Z* ≥ 1.96, and hence it is also called global FDR. pFDR is also viewed as the Bayesian posterior probability that a significant test belongs to a true null hypothesis [33, 37]. Bayesian interpretation of FDR serves as a connection between the frequentist and Bayesian theory.

Efron et al. maneuvered the Bayesian concept of the global FDR a bit more intricately to overcome most of the aforementioned problems [31, 37, 38]. It estimates local false discovery rate by approximating a fully hierarchical Bayesian model, in which the priors are common for all tests and estimated from the observed data assuming empirical or theoretical null distribution and thus the dependence structure is incorporated. The local FDR is computed as the posterior probability that a hypothesis is null given an observed statistic, *P*(*null*|*Z* = *z*). The large-scale testing leverages this local FDR inference for any observed statistic, which helps in judging the significance of the observed statistic on its own without referring to the tail area, e.g., *Z* ≥ 1.6. This approach allows us to frame better questions and confront the significance issue in a direct manner rather than by relying on the indirect reasoning that lies at the core of all p-valued methods.

The locFDR procedure was developed for microarray data analysis involving thousands of genes tested for multiple strain effects that result in millions of simultaneous tests. As high-dimensional PLV data acquired from EEG shares similar issues of large-scale testing, where the distribution of synchrony measures depends on time, frequency, and spatial location, we sought to investigate whether Efron’s locFDR method can serve as an effective tool for testing the significance of PLV inference. The procedure works well as we have seen both in the experimental data and simulation data examples. The locFDR inference with experimental data was superior than FDRBH, hFDR and ODP while conventionally controlling FDR at 5% cutoff; it detected more significant pairs than hFDR, whereas FDRBH and ODP did not detect any pair. At a more lenient cutoff of 10%, the Empirical Bayesian inference converges with the results from frequentist hFDR and ODP methods in the 22–34 Hz frequency band and 300–550 ms post stimulus time interval for the inefficient than efficient condition (Fig 4). The results from FDRBH at 10% cutoff showed only 10 pairs. The practice of setting Type I error threshold at 5% dates back to classic single testing, specifically using family-wise error rate (FWER) controlling procedures. For FDR, there are no such standards, though most existing FDR controlling methods impose the need to fix an acceptable FDR level before any data are seen. The locFDR quantity itself is a measure of significance of Type I error control, and should be reported for each interesting signal concerning the experimental hypothesis. Researchers may use their discretion to select a threshold, which is appropriate for their research domain, e.g., for microarray inference, 10% or 20% thresholds are acceptable [37]. The FDR curve that shows power of the test as a function of FDR (Fig 2) can serve as a reference for a reasonable choice.

In the simulation analysis, locFDR detected more discoveries than ODP when the proportion of discoveries was small, at *π*_1_ = .08 and *π*_1_ = .16. This situation typically describes our research problem with a high dimensional PLV data, where very few pairs are expected to contain the interesting or significant effects. Recall here that *π*_1_ was estimated to be 0.1 for the experiment data. Although we did not examine the robustness of each method against PLV strength directly (by varying magnitude of PLV for designated true positives across different simulations), we effectively evaluate robustness by varying the number (or ratio) of true positives, etc. (see Fig 1), which provides an indication of robustness. The reasoning is that if the strength of PLV is decreased, then (naturally) we expect fewer (uncorrected) detections, on which the correction methods are based, since a detection is obtained by the underlying (z) statistic. Hence, the robustness (of the correction method) can be simply evaluated by varying the number (ratio) of true positives, etc., as shown in the figure.

As locFDR is computed empirically from the null and mixture densities, it accommodates both asymmetric and symmetric distributions equally effectively. In contrast, the performance of ODP depends on the homogeneity of the dependence structure among hypotheses, and thus it is expected to perform better with asymmetric data. Asymmetry is implied when most PLV effects are in a particular direction, i.e. they are either mostly positive or mostly negative, which indeed seems to be the case with our experimental data that showed more significant PLV in inefficient-efficient contrast than in efficient-inefficient contrast (Fig 4). The simulated data was also asymmetric (with greater proportion of positive PLV effects than negative PLV effects) as it was generated using the parameters obtained from a real PLV dataset. For a comparison, we ran the simulation with an induced symmetry by assuming both positive and negative PLV effects in similar proportions. We observed that symmetry reduced the number of detections by ODP considerably when the proportion of true discoveries was smaller than 25%. Symmetry did not affect the performance of locFDR and hFDR (results not shown here).

In the experiment analysis, hFDR, ODP, and locFDR offered almost similar qualitative results. While the inference obtained from all the three methods are comparable, each method has its own advantages. hFDR approach is more amenable for datasets that are naturally hierarchical and is particularly powerful when the synchrony effects are concentrated in a few families of hypotheses. However, the PLV effects may not be bound to a given dimension (or a few families). In such cases, ODP and locFDR may be more powerful, both of which explicitly account for the inherent dependence structure by sharing the common information from all the tests. They both assign a direct significance measure of FDR to each of the test, eliminating the need to determine a cutoff. Their disadvantage over hFDR is that they work well only with large number of hypotheses (at least several hundred). The local FDR is more accurate than ODP for estimating FDR. The significance in ODP is determined using global FDR from the entire tail area, which gives an aggregated estimate. The locFDR procedure estimates the local FDR, which is determined for each test using the specific point in the tail, and it is more accurate. ODP requires computing a new statistic, which restricts its application. In comparison, locFDR is more widely applicable, e.g., to normal, chi-square, t-static, and F distributions, which are commonly used in our research domain [38, 39]. If the distribution is uncertain, the null can be defined empirically from the data. In this article, we have assumed the normal distribution, which holds reasonably well in the case of normalized PLVs [28, 40], and hence it can be tested with z-test or t-test. In the case of un-normalized absolute PLVs, the Gaussian distribution is unlikely to hold as they are bounded between 0 and 1. According to a recent study [11], von Mises and circularly symmetric Gaussian distributions are adequate for absolute PLV data and can be tested by Chi-square statistic for goodness of fit of these distrubutions.

Recent research in brain dynamics has established a number of methods for quantifying functional connectivity in EEG studies (see [1, 3] for a review of some of these methods). However, no standard method exists to justify the statistical significance of the connectivity inference from these methods, specifically when there is no a priori knowledge about the expected region of synchrony. Therefore, our article, which illustrates locFDR method for computing the false discovery rate associated with each PLV test, would benefit functional connectivity research.

While the Bayesian inference for EEG synchrony is available in the dynamic causal model of SPM [21], it eschews the frequentist multiple comparison issue. Friston et al. suggest that reporting of the posterior probability of activation is sufficient for Bayesian inference and to avoid the frequentist multiple comparison issue [20]. However, if we want to control wrong labeling of voxels (or pairs of EEG signals in our problem) across tests, the multiple comparison correction is still necessary on Bayesian posterior probabilities [41]. The proposed Empirical Bayes method brings together both the frequentist and Bayesian approaches, in the sense that we can report and control the estimated local FDR at each test, which is defined as the posterior probability that a test belongs to null group, and controlling it. The locFDR approach is readily applicable to the other available methods for measuring synchrony, e.g., mutual information, generalized synchronization [42], single-trial phase locking [43], structural synchrony [13], empirical mode detection PLV [44], phase resetting [45, 46], a method based on Cohens class of time-frequency distributions [47], and a recently published graph partitioning method for modeling brain connectivity [48].
